# Differential Risk of Cognitive Impairment across Paid and Unpaid Occupations in the Middle-Age Population: Evidence from the Korean Longitudinal Study of Aging, 2006–2016

**DOI:** 10.3390/ijerph17093124

**Published:** 2020-04-30

**Authors:** Woojin Chung, Roeul Kim

**Affiliations:** 1 Department of Health Policy and Management, Graduate School of Public Health, Yonsei University, Seoul 03722, Korea; wchung@yuhs.ac; 2 Institute of Health Services Research, Yonsei University, Seoul 03722, Korea; 3 Labor Welfare Research Institute, Korea Workers’ Compensation and Welfare Service, Seoul 07254, Korea

**Keywords:** cognitive impairment, occupation, unpaid work, middle-age population, gender, longitudinal studies, South Korea

## Abstract

To examine and quantify the risk of cognitive impairment across a variety of occupations including unpaid work in a middle-age population using the dataset of a nationally representative longitudinal survey. A total of 20,932 observations of 5865 subjects aged 45–64 were obtained from six waves of the Korean Longitudinal Study of Aging (2006–2016). A dichotomous outcome variable was constructed on the basis of the Korean Versions of the Mini-Mental State Examination scores, and occupations were grouped into 10 occupation categories, including unpaid housekeepers. Socio-demographics, lifestyle, and medical conditions were used as covariates in mixed logistic regression models. Adjusted odds ratios and predicted probabilities of cognitive impairment were computed and adjusted for a complex survey design. In longitudinal models with all studied covariates, the risk of cognitive impairment differed significantly across occupation categories, but the association of occupation with the risk of cognitive impairment was the same between genders. In terms of the predicted probability, the risk of cognitive impairment in the unpaid housekeepers’ category (11.2%, 95% confidence interval (CI): 10.4% to 11.9%) was the highest among occupation categories, being three times higher than in the professionals’ and related workers’ category (3.7%, 95% CI: 1.6% to 5.7%). Public policies based on studies of the risk of cognitive impairment across different occupations in the middle-age population should be designed so as to prevent cognitive impairment in the middle-age population as well as their older life stages, particularly targeting high-risk groups such as people engaged in unpaid domestic and care activities.

## 1. Introduction

Cognitive impairment imposes considerable socioeconomic burdens on society that tend to increase with population aging; for example, the current national dementia cost to South Korea (hereafter, Korea) is estimated to be approximately 12 billion USD, which is approximately 0.9% of the Korean national gross domestic product (GDP). This cost is expected to double every 10 years and will reach approximately 93 billion USD by 2050, which is approximately 3.8% of the expected Korean national GDP [1]. In 2015, the age-standardized incidence of Alzheimer disease and mild cognitive impairment among Koreans was reported to be 7.9 and 28.1 cases per 1000 person-years, respectively [2]. Risk factors of cognitive impairment include age [3], family history [4], education [5], occupation [6], brain injury [7], exposure to pesticides or toxins [8], physical inactivity [9], and chronic conditions such as Parkinson disease, heart disease, stroke, and diabetes [10].

In particular, an individual’s occupation before retirement has been reported to be strongly associated with his/her risk of cognitive impairment after retirement. Studies of cognitive function before and after retirement in the United States have shown that because working in an occupation with a higher level of mental demands needed a higher level of cognitive functioning before retirement, it likely results in a slower rate of cognitive decline after retirement [11,12]. A study in Taiwan found that elderly people who had been forestry, fishing, and craft workers were at higher risk for cognitive impairment than former legislators, business executives, and managers [13]. Moreover, a few in-depth studies have been conducted in middle-age populations on the relationship between occupation and cognitive impairment. For example, a study conducted in the Netherlands showed that primary and secondary school teachers have better working memory and verbal fluency abilities than other occupations, even when matched for age, gender, occupation, and education [14]. In a study in the United States, greater occupational complexity was associated with decreased hippocampal volume and increased whole-brain atrophy in a middle-aged cohort. These results suggest that in people at risk for Alzheimer disease, occupational complexity may confer resilience against the adverse effects of neuropathology on cognition [15].

Although previous studies have contributed greatly to illuminate the relationship between occupation and cognitive impairment, they show several limitations: A lack of nationally representative samples, a reliance on cross-sectional data, limited categories of occupations, and insufficient examination of potential gender differences in the relationship between occupation and cognitive function. Moreover, because only older populations were analyzed, except in a few studies, it is difficult to understand how the relationship between occupation and cognitive impairment in the middle-age population would operate. Above all, no study attempted to examine both unpaid work and a variety of paid occupations, although unpaid work contributes to between one-third and one-half of all valuable activities in the Organization for Economic Cooperation and Development (OECD) member countries and emerging economies [16]. 

In this study, therefore, we aimed to address a substantial gap in the literature and to examine the association between paid or unpaid occupations and the risk of cognitive impairment in the middle-age population using a Korean national panel survey. Meanwhile, Korea’s per capita GDP, at $34,549 as of 2016, meets the criteria of a developed country by any reasonable standard. The GDP per capita of Korea in 2019 was $33,320. In 2019, life expectancy for Korea was an impressive 82.92 years; the total population was about 51.2 million people [17]. Korea has one of the world’s highest educated labour forces among OECD member countries [18]. In addition, Korea is one of the top performing OECD member countries in reading literacy, mathematics, and science, with an average score of 519, compared with the OECD average of 493, placing it ninth in the world [19]. The unemployment in Korea as of April 2019 was 4.8 percent, which is comparatively low compared to most OECD member countries [20]. Because Korea is experiencing much more rapid population aging than any other OECD member country [21], the results of this study would be useful for other countries facing population aging to understand the association between occupation and the risk of cognitive impairment in the middle-age population and to prepare appropriate policies to reduce the socioeconomic burden imposed by the high prevalence of cognitive impairment accompanying population aging. 

To achieve the aim, we tested two hypotheses in this study: (A) The risk of cognitive impairment is the same across occupation categories and (B) the association between occupation and the risk of cognitive impairment is the same between genders. A number of considerations led us to test the two hypotheses in each of three cases: Case 1, analyses of individuals at a certain time (cross-sectional analysis); Case 2, analyses adjusted for temporal correlation between observations within an individual (longitudinal analysis with no covariate); and Case 3, analyses adjusted both for temporal correlation between observations within an individual and for all studied covariates (longitudinal analysis with all studied covariates). To summarize, we investigated six hypotheses from (1-A) to (3-B) and tested each. 

In addition, for expository convenience, when estimating the predicted probability of cognitive impairment when an individual was engaged in a specific occupation, we attempted to quantify the risk of cognitive impairment and compare the risk across paid and unpaid occupations. 

## 2. Materials and Methods

### 2.1. Data Source and Study Sample

We used a sample drawn from the first six waves of the Korean Longitudinal Study of Aging (KLoSA) survey, a nationally representative non-institutionalized, civilian population survey conducted biennially from 2006 to 2016. In order to facilitate the development of policies for an aging society, the KLoSA survey used a stratified, multistage, clustered probability sampling design to collect data on Koreans aged 45 years and over. The KLoSA survey was carried out on behalf of the Korea Employment Information Service under the Korean Ministry of Employment and Labor. In the first baseline survey in 2006, 10,254 individuals in 6171 households were interviewed using computer-assisted personal interviewing. The KLoSA survey included information regarding labor/employment-related, socio-demographic, lifestyle, and health-related characteristics. Detailed information about the survey design and characteristics is available at the KLoSA website (https://survey.keis.or.kr/eng/klosa/klosa01.jsp).

We included participants surveyed at baseline (i.e., the first wave) in 2006 and then surveyed at least once at the later surveys, collecting 22,871 observations for respondents aged 45–64 years. From these we excluded: (1) Noncontact, refusal, or death (688 observations); (2) diagnosis with intellectual disabilities, organic brain diseases, or psychiatric treatments (590 observations); and (3) nonreport of the Korean Mini-Mental State Examination (K-MMSE) scores (661 observations). The final study sample comprised 5865 participants at baseline and 20,932 observations with an average of 3.52 observations per participant (standard deviation = 1.97, range = 1–6). When the KLoSA survey was taken, informed consent was obtained from all participants in line with the ethical principles of the Declaration of Helsinki. The Yonsei University Health System Institutional Review Board approved this study (Y-2019–0178).

### 2.2. Measurements

To determine cognitive function, we used the Korean version of the Mini-Mental State Examination (K-MMSE) for cognitive function [22,23]. The K-MMSE includes 11 items divided into two sections, which involve both verbal and written responses to questions measuring orientation in time and space, memory, and attention. The total K-MMSE score ranges from 0 to 30, with higher scores representing better cognitive function. Defining cognitive impairment as a K-MMSE score less than 24 [24,25], we constructed a dichotomous outcome variable with a value of 1 (cognitive impairment, K-MMSE score < 24) and 0 (no cognitive impairment, K-MMSE score ≥ 24). 

Regarding occupations, we grouped participants who had a job in the labor market into nine categories with reference to the Korean Standard Classification of Occupations, a Korean version of the International Standard Classification of Occupations of the International Labour Organization. The categories were (1) managers; (2) professionals and related workers; (3) clerks; (4) service workers; (5) sales workers; (6) skilled agricultural, forestry, and fishery workers; (7) craft and related trades workers; (8) plant, machine operators, and assemblers; and (9) elementary workers and armed forces.

In particular, we paid special attention to unpaid work around the world [16,26,27]. People who engage in unpaid work likely conduct unpaid domestic and care work, from cooking and housekeeping to looking after children and ill, elderly, or disabled relatives. In order to include unpaid works in our analysis, we grouped participants who had no job in the labor market (homemakers, unemployed, or retired) as a category of unpaid domestic and caring activities (shortly, unpaid housekeepers). As a result, we considered 10 paid and unpaid occupational categories (nine paid occupations plus one unpaid occupation) as the variable of interest.

As for potential covariates, we incorporated 13 characteristics of participants. Socio-demographic characteristics were: Gender (man and woman), age, marital status (married and nonmarried, where nonmarried included never married, separated, widowed, or divorced), residential area (urban area and rural area), educational attainment (elementary school or less, middle school or high school, and college or higher, according to the highest level of formal education completed), household income (lower half, higher half, and unreported), and housing tenure (house owner and house renter). For household income, for each wave, we adjusted household size by the square-root equivalence scale [28] and divided it into two groups at its median value, and we kept participants not reporting their household income (5.8% of participants at baseline) as a separate group in order not to lose their valuable information. In addition, characteristics about lifestyle and medical conditions were: Smoking (smoker and nonsmoker), alcohol drinking (alcohol drinker and non-alcohol drinker), routine physical exercise activity (active and not active), obesity (obese and not obese), chronic disease (no and yes), and depressive symptom (no and yes). We assessed routine physical exercise activity according to information on participants’ response to a survey question whether the participants engaged in any physical exercise at least once a week for the sake of their own health. We defined obesity as the body mass index of at least 25 on the basis of the revised Asia-Pacific criteria by the World Health Organization of the Western Pacific Region [29]. We defined chronic disease based on self-reported answers to survey questions whether a chronic disease (hypertension, diabetes, stroke, angina, myocardial infarction, chronic pulmonary diseases, and any type of cancer) had been diagnosed by a physician. We defined depressive symptom as a score of four or more on the 10-item short form of the Center for Epidemiologic Studies Depression Scale (CES-D10) [30,31].

### 2.3. Statistical Analysis

We performed the following statistical analyses to test the hypotheses for each of three cases.

For Case 1 (a cross-sectional analysis), we used observations at baseline (Wave 1). To test whether the risk of cognitive impairment was the same across occupation categories (Hypothesis 1-A), we estimated prevalence rates of cognitive impairment for each occupation category and compared them across occupation categories with a chi-squared test. Then, to test whether the association between occupation and the risk of cognitive impairment was the same between genders (Hypothesis 1-B), we constructed a simple logistic model with an occupation variable, a gender variable, and an interaction effect term between these two variables and examined the statistical significance of the interaction effect term using the Wald test.

For Cases 2 and 3 (longitudinal analyses), we used all observations from all waves. Because observations in a longitudinal dataset are likely to be temporally correlated within the same participant, we employed a mixed logistic regression model with two levels. Indeed, the null model showed a considerable degree of intraclass correlation (0.651, 95% confidence intervals: 0.608 to 0.691). In addition, we took into consideration that multilevel model estimation requires weights at each level as well as their rescaling, for otherwise the estimation is likely to cause bias in parameter estimates, especially for small samples [32] and for multilevel logistic regression models [33]. We, therefore, rescaled the conditional weights at level 1 of the data hierarchy to normalize these conditional weights to sum to within-cluster sample sizes [32].

Regarding Case 2 (a longitudinal analysis with no covariate), we constructed two models: A model with only the occupation variable as an independent variable to test whether the risk of cognitive impairment was the same across occupation categories (Hypothesis 2-A), and another model with an occupation variable, a gender variable, and an interaction effect term between these two variables to test whether the association between occupation and the risk of cognitive impairment was the same between genders (Hypothesis 2-B). Regarding Case 3 (a longitudinal analysis with all studied covariates), we added all studied potential covariates to the two models of Case 2 and tested the equality of the risk of cognitive impairment across occupation categories (Hypothesis 3-A) and the equality of the association between occupation and the risk of cognitive impairment between genders (Hypothesis 3-B). 

For the multivariable analyses performed for Case 3, we took several steps to find appropriate model specifications. First, we continued to re-categorize each categorical variable and defined each reference category differently, so that throughout all models, the values for the variance inflation factor were <2.08, implying no strong multicollinearity, and *p*-values based on the Hosmer–Lemeshow statistic were >0.745, showing no evidence of lack of goodness-of-fit. Then, using Pseudo-Akaike’s information criterion as a measure of the goodness-fit of a mixed model, we chose a random intercept model along with an unstructured diagonal covariance structure. We found all covariance parameter estimates for each model to be significant (*p* < 0.0001), which suggests that each model was adjusted for a considerable degree of correlations between observations within a participant.

To explore the degree of the risk of cognitive impairment that an individual engaged in a specific occupation faces, we estimated a participant’s predicted probability of cognitive impairment (and its 95% confidence intervals) if the participant were engaged in a particular occupation, all other characteristics were held constant at the participant’s own values, and then it was tested whether the predicted probability was the same for a particular occupation category and the reference occupation category, that is, unpaid housekeepers. In addition, this study tested whether the predicted probability for a particular occupation category was the same between genders.

For analysis, we considered all characteristics to be time-dependent and conducted all estimation processes with a complex sampling design. For both the prevalence rates of cognitive impairment and their associations with each characteristic, we estimated odds ratios (ORs) and their 95% confidence intervals (CIs), setting *p*-values < 0.05 (two-tailed) to be statistically significant. We used SAS 9.4 (SAS Institute, Cary, NC, USA) and STATA 15 (StataCorp, College Station, TX, USA) to carry out all statistical analyses.

## 3. Results

Table 1 shows the sample characteristics at the baseline (Wave 1). The mean cognitive function score and age were 27.4 and 54.0 years, respectively. The highest proportion of participants for each characteristic was found in the following corresponding categories: woman; married; residing in an urban area; middle school or high school graduate; lower half of household income; house owner; nonsmoker; non-alcohol drinker; active routine physical exercise; not obese; no chronic disease; or no depressive symptom.

Table 2 presents the prevalence of cognitive impairment across occupation categories at baseline and the distribution of observations across occupation categories by wave. For the prevalence rate of cognitive impairment at baseline, the overall rate was 8.6% (95% confidence interval (CI): 7.9% to 9.3%), and the rate in the unpaid housekeepers category (12.7%, 95% CI: 11.4% to 14.0%) was 5.5 times higher than the rate in the professionals and related workers category (2.3%, 95% CI: 0.5% to 4.1%). Based on the Rao–Scott chi-squared test, the rate was significantly different across occupation categories (*p* < 0.0001). In addition, the Wald test showed no significant interaction effect term between occupation and gender variables (*p* = 0.259). Therefore, concerning Case 1 (a cross-sectional analysis), we may conclude that Hypothesis 1-A is not true, suggesting that the risk of cognitive impairment varied across occupation categories; but Hypothesis 1-B is true, suggesting that the association between occupation and the risk of cognitive impairment is the same between genders.

Table 3 shows the results of the longitudinal analyses of the associations of occupation categories with cognitive impairment. According to the model with no covariate, relative to the unpaid housekeepers’ category, the OR of cognitive impairment for each of most other occupation categories was much lower and significantly different. In particular, the professionals and related workers’ (0.05, 95% CI: 0.02 to 0.11) and the clerks’ categories (0.19, 95% CI: 0.11 to 0.32) revealed the two lowest ORs of cognitive impairment. By contrast, the elementary workers and armed forces category (0.64, 95% CI: 0.51 to 0.81) and the skilled agricultural, forestry, and fishery workers category (0.75, 95% CI: 0.54 to 1.06) had the two highest ORs of cognitive impairment, of which the OR of the skilled agricultural, forestry, and fishery workers category did not significantly differ from that of the unpaid housekeepers category (*p* = 0.103). Meanwhile, the Wald test showed that the interaction effect term between occupation and gender was significant (*p* = 0.020). Therefore, concerning Case 2 (a longitudinal analysis with no covariate), after adjusting for temporal correlations between observations within an individual, it appears that Hypothesis 2-A is not true, implying that the risk of cognitive impairment varies across most occupation categories; and Hypothesis 2-B is not true, so that the association between occupation and the risk of cognitive impairment differs between genders. 

In the model with all-studied covariates, compared to the unpaid housekeepers’ category, the ORs of cognitive impairment for every other occupation category increased differently from the ORs in the model with no covariate, except for its decrease in the skilled agricultural, forestry, and fishery workers’ category. Among these, the OR of cognitive impairment was very low in the professionals and related workers’ category (0.19, 95% CI: 0.09 to 0.40) and the sales workers’ category (0.48, 95% CI: 0.34 to 0.69). By contrast, its OR was very high in the managers’ category (0.71, 95% CI: 0.44 to 1.16) and the elementary workers’ and armed forces’ category (0.69, 95% CI: 0.55 to 0.86), although the OR of cognitive impairment for the managers category did not differ from that of the unpaid housekeepers category. Meanwhile, according to the Wald test, the interaction term between occupation and gender was significant (*p* = 0.165). Therefore, concerning Case 3 (a longitudinal analysis with all-studied covariates), after adjusting both for temporal correlation between observations within an individual and for all-studied covariates, we may conclude that Hypothesis 3-A is not true, suggesting that the risk of cognitive impairment varies across most occupation categories; but Hypothesis 3-B is true, which suggests that the association between occupation and the risk of cognitive impairment is the same between genders, thereby indicating no need for this model to be stratified by gender.

**Figure 1:**
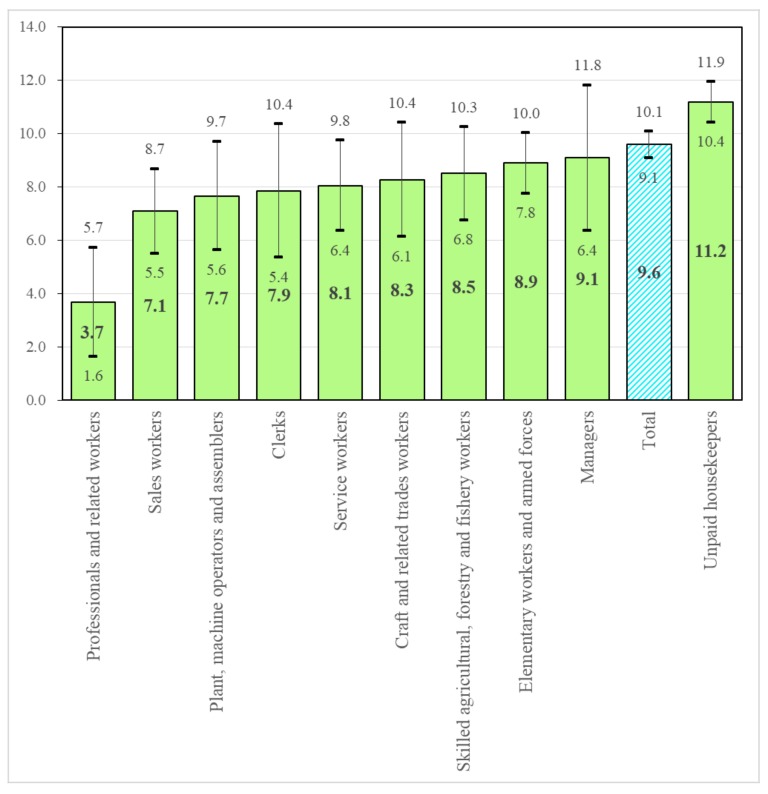
Predicted probability (%) and its 95% confidence interval of cognitive impairment by occupation category.

Based on the results obtained from the model of Case 3 (a longitudinal analysis with all-studied covariates), we estimated the predicted probabilities of cognitive impairment for each occupation category and their 95% CIs using the delta method and arranged them from the lowest value to the highest in Figure 1. In terms of the predicted probabilities of cognitive impairment, the unpaid housekeepers’ category showed the highest risk of cognitive impairment (11.2%, 95% CI: 10.4% to 11.9%) among all occupation categories except for the managers’ category, in sharp contrast with the professionals and related workers’ category, which has the lowest risk (3.7%, 95% CI: 1.6% to 5.7%). Meanwhile, the Wald test showed that the predicted probability for a particular occupation category was the same between genders (*p* > 0.391 for each occupation category).

## 4. Discussion 

In this study, we investigated the association between paid or unpaid occupation and the risk of cognitive impairment in the middle-age population. Based on the results obtained from a model adjusting both for temporal correlation between observations within an individual and for all studied covariates, the three main results are as follows: First, the risk of cognitive impairment varied across paid and unpaid occupations; second, the associations between occupation and the risk of cognitive impairment were equal between genders; third, the risk of cognitive impairment was the highest in the unpaid housekeepers category among all studied occupation categories, as it was shown that the predicted probability of having cognitive impairment in the unpaid housekeepers’ category (11.2%) was three times higher than that in the professionals and related workers’ category (3.7%).

Our results that the risk of cognitive impairment occupation differed across paid and unpaid occupations and that the professional and related workers category had the lowest risk of cognitive impairment among all occupation categories seem consistent with the results of previous research that cognitive performance was significantly related to occupation characteristics such as occupational complexity and patterns of occupational demands [11,13,14,34,35,36]. Because the professional and related workers’ category includes jobs subject to high mental requirements, our finding is in line with the results of earlier studies that individuals who have occupations with high mental requirements show better performance of cognitive ability [6,35,37].

Importantly, some studies have revealed that occupations in which individuals had been engaged in their middle-age life stages influenced the time of onset for cognitive impairment in their older life stages [11,12]. A study using longitudinal data from the Maastricht Aging study conducted in the Netherlands found that individuals with mentally demanding jobs had lower risks of developing cognitive impairment than other individuals [35]. Similarly, another study drawing on the Leukoaraiosis and Disability study in Europe showed that cognitively demanding occupations such as white-collar, professional, or managerial work was related to slower decline in working memory and immediate memory recall [38].

These results may be partly due to the “use it or lose it” perspective on cognitive aging [39,40]. The cognitive reserve hypothesis suggests that individuals engaged in more mentally demanding work will not experience cognitive decline or will experience slower cognitive decline than those engaged in less mentally demanding work [11]. The process whereby mental stimulation at work affects cognitive function either involves a direct neuroprotective effect or an effect or brain reserve, thereby postponing symptoms of cognitive decline rather than significantly affecting the underlying pathology [41,42]. In this interpretation, brain reserve is considered subject to further changes during adulthood, instead of being fully developed in early life and stable thereafter [35].

Our findings showed that in the middle-age population, unpaid housekeepers face a risk of cognitive impairment three times higher than do their counterparts in the professions and related occupations, being in line with the results of previous studies that individuals engaged in activities that made significant loads on their cognitive skills showed greater maintenance or improvement of their cognitive abilities than did their counterparts who were exposed to less complex environments with minimal cognitive loads [43,44,45]. In addition, there is ample evidence that unemployment leads to psychological problems and distress, which in turn can lead to cognitive impairment [46,47]. A paid job assures economic security as well as a social network related to life opportunities and social support; it may also help maintain physical and intellectual stimulation. Unpaid work such as childcare, housework, and home maintenance is more physically demanding but less intellectually stimulating, so unpaid work is less protective of cognitive function [47]. Either continuing to work or to perform volunteer work seems to reinforce cognitive function [48]. Meanwhile, a study of the elderly using cross-sectional data from Zhejiang Province in China showed that the risk of cognitive impairment was highly associated with primary lifetime occupation as housewives [36].

With regard to gender differences in the association between occupation type and the risk of cognitive impairment, this study revealed no differences in this association between genders in the middle-age population. In line with this, several studies found no gender differences with regard to cognitive impairment in the middle-age and elderly, excluding the oldest old (≤80) [49,50]. A study using 2347 samples from the Doetinchem Cohort Study showed that a composite risk score comprising unhealthy lifestyle and relatively poor health in midlife is significantly associated with a worse course of cognition 10 years later. These associations were for the most part unrelated to gender differences [49]. Analyzing longitudinal studies of the past decade (2001–2011), a study observed that gender does not determine the rate of cognitive decline in the 60–80-year age group and that cultural and lifestyle differences between the genders are determining factors in the severity of cognitive decline. In certain cultures, women have a lower educational level, are less engaged in professional occupations, and have lower social participation than men have. The contextual and cultural factors related to gender, rather than gender itself, determine cognitive decline [50]. Therefore, a potential explanation for our results relates to changing societal conditions such that men and women participating in the same occupations have similar levels of economic security as well as similar social networks and social support. Increased exposure to cognitive stimulation, economic prosperity, health improvements, and changes in average family size seem to be particularly advantageous for women over time [51].

Meanwhile, referring to information provided by OECD, in 2018, the ratio of the people who have not participated in gainful employment in the working age population (15 to 64 years) among 36 OECD member countries was as high as 30% on average, ranging from 14.9% in Iceland to 48.0% in Turkey. In particular, the ratio for women relative to men showed a higher average value, 35.1%, with a range from 17.5% in Iceland to 67.1% in Turkey [52]. Though drawing policy suggestions based on the results of this study needs substantial caution, we may suggest that in the face of a potentially increasing prevalence of cognitive impairment accompanied with population aging, rational policies should be designed and implemented to reduce the risk of cognitive impairment from the middle-age life stage, particularly targeting its high risk groups such as people who are engaged in unpaid domestic and caring activities (homemakers, unemployed, or retired). Of course, in the future, studies like the present study in Korea should be conducted in each country whose population is aging or aged, taking country-specific characteristics into full and detailed consideration.

The present study has several strengths compared to previous studies of the association between occupation and the risk of cognitive impairment. First, we analyzed a nationally representative longitudinal data with 10 types of occupations, including one unpaid occupation. Second, we focused on the risk of the cognitive impairment in the middle-age population, which will be elevated in the elderly population. Third, we closely examined potential gender differences in the association. Fourth, we conducted in-depth, three-dimensional analyses (a cross-sectional analysis, a longitudinal analysis with no covariate, and a longitudinal analysis with all-studied covariates). Finally, to the best of our knowledge, the current study is the first to quantify the risks of cognitive impairment and compare them across a variety of occupations in terms of the predicted probability of cognitive impairment.

However, this study has several limitations. First, cognitive function was measured by the K-MMSE on the basis of respondents’ self-reports, without other clinical assessments such as a clinical dementia rating scale or neuropsychological battery [53]. It may thus not reflect actual capabilities or adequately address cognitive functional problems. However, the Mini-Mental State Examination (MMSE) is a convenient alternative measure to detailed neuropsychological testing [54]. Its use allowed us to evaluate cognitive changes in a large number of subjects [53,55]. Future studies would benefit from the use of more comprehensive neuropsychological or other cognitive indices to evaluate cognitive functions. Second, we could not estimate premorbid intelligence, which is a predictor of MMSE scores along with age, education, and occupation [56], as the dataset does not provide such information. Future research should consider premorbid intelligence when investigating the risk of cognitive impairment across diverse occupations, including unpaid work, in the middle-age population. Third, we could not include variables that are possibly related to cognitive impairment such as job turn-over, duration of each occupation, and social support, for the dataset does not provide such information. Meanwhile, a study of people aged 60 years or more in Korea found that longer work duration in young adulthood might be a positive contributor to cognitive function in old adulthood [57]. Fourth, because some occupation categories lacked a number of observations, it was difficult to correctly explore the association between the occupation and the risk of cognitive impairment. For example, the reason that the predicted probability of cognitive impairment was not different between the managers’ category and the unpaid housekeepers’ category might be partly because the number of observations in the managers’ category was the smallest (3.2%, as shown Table 2) of all occupation categories. Future research needs a sufficient number of observations for every occupation category. Finally, we did not break down the unpaid housekeepers’ category because in the present study, we intended to include all people who are engaged in unpaid domestic and caring activities in one category, whether they are homemakers, retired individuals, students, or unemployed people. However, if the category is further categorized, it is possible that the association of occupation with the risk of cognitive impairment would be found to differ by gender, in which case all analyses would take gender into account. Our next study will address this issue in detail.

## 5. Conclusions 

This is the first study to investigate and quantify the risk of cognitive impairment across a diversity of occupations including unpaid work in the middle-age population using the dataset of a national longitudinal study. In the present study, we found that the risk of cognitive impairment varied across occupations but its association with occupation was the same between genders in Korea. In terms of predicted probability, the risk of cognitive impairment was three times higher in the unpaid housekeepers’ category than in the professionals and related workers’ category, implying that a greater intellectual stimulation in the workplace might be beneficial to cognitive function. From a policy perspective, public policies based on studies of the risk of cognitive impairment across different occupations in the middle-age population should be designed and implemented in order to contribute to preventing the risk of cognitive impairment in these age groups as well as in their later life stages. In particular, we highlighted targeting high risk groups such as people engaged in unpaid domestic and caring activities. Further research is needed to investigate whether these results and corresponding suggestions are valid in other settings in terms of either socio-culture or economic development.

## Figures and Tables

**Table 1:** Sample characteristics at baseline.

Characteristics	Number or %
Cognitive function ^a^: Mean (SD) ^b^, median	27.4 (3.1); 28.0
Male	44.7%
Age, years: Mean (SD) ^b^; median	54.0 (5.9); 54.0
Married ^c^	88.5%
Reside in a urban area	81.5%
Educational attainment	
Elementary school or less	29.4%
Middle school or high school	56.8%
College or higher	13.8%
Household income ^d^	
Lower half	47.5%
Higher half	46.7%
Unreported	5.8%
House owner	72.2%
Smoker	22.2%
Alcohol drinker	45.0%
Active routine physical exercise	43.3%
Obese ^e^	24.7%
Have chronic disease ^f^	28.9%
Have depressive symptom ^g^	22.2%
Number of observations	5865

^a^ Cognitive function was based on the Korean Mini-Mental State Examination scores. ^b^ SD denotes standard deviation. ^c^ Nonmarried includes never married, separated, widowed, or divorced. ^d^ Household income was adjusted for household size for each wave. ^e^ Obese was defined as the body mass index of at least 25. ^f^ Chronic diseases include hypertension, diabetes, stroke, angina, myocardial infarction, chronic pulmonary diseases, and any type of cancer. ^g^ Depressive symptom was defined as a score of 4 or more on the 10-item short form of the Center for Epidemiologic Studies Depression Scale.

**Table 2:** The prevalence of cognitive impairment by occupation category at baseline, and the distribution of observations across occupation categories by wave.

Occupation	Prevalence (%)		Distribution (%)						
	Rate	(95% CI)	Wave 1	Wave 2	Wave 3	Wave 4	Wave 5	Wave 6	Overall
Unpaid housekeepers ^a^	12.7	(11.4–14.0)	47.1	41.8	38.8	40.9	41.8	41.1	42.6
Managers	2.5	(0.7–4.4)	5.7	3.5	3.0	2.9	3.0	3.2	3.9
Professionals and related workers	2.3	(0.5–4.1)	3.3	5.1	5.2	5.0	4.6	4.5	4.5
Clerks	2.4	(0.7–4.0)	5.0	4.5	4.4	3.8	3.7	3.3	4.3
Service workers	6.5	(3.9–9.1)	6.2	7.6	7.8	7.5	7.7	8.6	7.3
Sales workers	3.3	(1.4–5.2)	6.5	7.5	7.5	6.9	6.4	6.4	6.9
Skilled agricultural, forestry and fishery workers	13.0	(9.0–17.1)	4.8	5.9	6.7	6.2	5.6	5.2	5.7
Craft and related trades workers	4.5	(1.9–7.1)	5.5	5.8	6.2	6.8	6.7	6.5	6.1
Plant, machine operators and assemblers	4.1	(1.7–6.4)	5.9	5.9	6.1	6.0	6.0	6.0	6.0
Elementary workers and armed forces	9.4	(7.0–11.8)	10.1	12.3	14.4	14.0	14.5	15.2	12.8
Chi-squared test, *p*-value		<0.0001							
Overall	8.6	(7.9–9.3)							
Number of observations	5865		5865	4418	3559	2928	2425	1737	20,932

Prevalence estimation and tests were carried out with a complex sampling design. ^a^ Unpaid housekeepers included the unemployed, the retired, or homemakers.

**Table 3:** Longitudinal analyses of the associations of occupation categories with cognitive impairment.

Characteristics	Model with No Covariate				Model with All-Studied Covariates		
	OR ^a^	(95% CI) ^b^		*p*	OR ^a^	(95% CI) ^b^	*p*
Occupation (Ref: Unpaid housekeepers)							
Managers	0.20	(0.12–0.33)		<0.001	0.71	(0.44–1.16)	0.176
Professionals and related workers	0.05	(0.02–0.11)		<0.001	0.19	(0.09–0.40)	<0.001
Clerks	0.19	(0.11–0.32)		<0.001	0.57	(0.34–0.94)	0.027
Service workers	0.40	(0.28–0.56)		<0.001	0.59	(0.42–0.83)	0.002
Sales workers	0.28	(0.19–0.41)		<0.001	0.48	(0.34–0.69)	<0.001
Skilled agricultural, forestry and fishery workers	0.75	(0.54–1.06)		0.103	0.64	(0.45–0.90)	0.011
Craft and related trades workers	0.34	(0.22–0.54)		<0.001	0.61	(0.40–0.94)	0.024
Plant, machine operators and assemblers	0.29	(0.19–0.44)		<0.001	0.55	(0.36–0.83)	0.005
Elementary workers and armed forces	0.64	(0.51–0.81)		<0.001	0.69	(0.55–0.86)	0.001
Man (Ref: Women)					1.10	(0.88–1.39)	0.394
Age (Ref: Mean value)					1.05	(1.04–1.07)	<0.001
Non-married ^c^ (Ref: Married)					1.19	(0.94–1.51)	0.138
Reside in a rural area (Ref: Reside in an urban area)					0.99	(0.80–1.23)	0.944
Educational attainment (Ref: Elementary school or less)							
Middle school or high school					0.22	(0.18–0.27)	<0.001
College or higher					0.12	(0.08–0.17)	<0.001
Household income ^d^, higher half (Ref: Lower half or unreported)					0.83	(0.72–0.96)	0.015
House renter (Ref: Owner)					1.13	(0.92–1.38)	0.255
Smoker (Ref: Non-smoker)					0.77	(0.60–0.99)	0.040
Alcohol drinker (Ref: Non-alcohol-drinker)					0.65	(0.54–0.78)	<0.001
Active routine physical exercise (Ref: Not active)					0.64	(0.55–0.75)	<0.001
Obese ^e^ (Ref: Not obese)					1.04	(0.87–1.23)	0.670
Have chronic disease (Ref: No)					1.24	(1.05–1.46)	0.011
Have depressive symptom^f^ (Ref: No)					2.22	(1.93–2.56)	<0.001

Effect of a continuous variable, age, was assessed as one unit offset from the mean. All values were estimated with a complex sampling design. All characteristics were considered to be time-dependent. ^a^ OR denotes odds ratio. ^b^ CI denotes confidence interval. ^c^ Nonmarried included never married, separated, widowed, or divorced. ^d^ Household income was adjusted for household size for each wave. ^e^ Obese was defined as the body mass index of at least 25. ^f^ Depressive symptom was defined as a score of 4 or more on the 10-item short form of the Center for Epidemiologic Studies Depression Scale.
